# Impact of PSA testing on secondary care costs in England and Wales: estimates from the Cluster randomised triAl of PSA testing for Prostate cancer (CAP)

**DOI:** 10.1186/s12913-023-09503-7

**Published:** 2023-06-09

**Authors:** Joanna C. Thorn, Emma L. Turner, Eleanor I. Walsh, Jenny L. Donovan, David E. Neal, Freddie C. Hamdy, Richard M. Martin, Sian M. Noble

**Affiliations:** 1 Bristol Medical School, Population Health Sciences, 1-5 Whiteladies Road, Bristol, BS8 1NU UK; 2 Bristol Medical School, Population Health Sciences, University of Bristol, Canynge Hall, 39 Whatley Road, Bristol, BS8 2PS UK; 3 Nuffield Department of Surgical Sciences, John Radcliffe Hospital, Oxford, OX3 9DU UK

**Keywords:** Cost-effectiveness analysis, Economic evaluation, Prostate cancer screening, Secondary care, Budget impact analysis

## Abstract

**Background:**

Screening men for prostate cancer using prostate-specific antigen (PSA) testing remains controversial. We aimed to estimate the likely budgetary impact on secondary care in England and Wales to inform screening decision makers.

**Methods:**

The Cluster randomised triAl of PSA testing for Prostate cancer study (CAP) compared a single invitation to men aged 50–69 for a PSA test with usual care (no screening). Routinely collected hospital care data were obtained for all men in CAP, and NHS reference costs were mapped to each event via Healthcare Resource Group (HRG) codes. Secondary-care costs per man per year were calculated, and cost differences (and population-level estimates) between arms were derived annually for the first five years following randomisation.

**Results:**

In the first year post-randomisation, secondary-care costs averaged across all men (irrespective of a prostate cancer diagnosis) in the intervention arm (*n* = 189279) were £44.80 (95% confidence interval: £18.30-£71.30) higher than for men in the control arm (*n* = 219357). Extrapolated to a population level, the introduction of a single PSA screening invitation could lead to additional secondary care costs of £314 million.

**Conclusions:**

Introducing a single PSA screening test for men aged 50–69 across England and Wales could lead to very high initial secondary-care costs.

## Background

The benefits and harms of population screening for prostate cancer continue to be debated [1, 2]. A simple blood test can measure blood levels of prostate specific antigen (PSA), a protein that when raised in the circulation indicates an increased risk of prostate cancer, and warrants further diagnostic investigations. However, while a European trial demonstrated that screening using repeated PSA testing reduces prostate cancer specific mortality [3], the UK CAP trial suggested that there is little prostate cancer specific mortality benefit of a single PSA screen after 10 years of follow-up [4], and overall the weight of evidence does not indicate that any potential mortality benefit outweighs the risks of overdiagnosis of indolent disease and of overtreatment [5]. Overdiagnosis and, consequently, overtreatment are substantial problems, leading to unnecessary, unpleasant side-effects for some men, and the benefit-harm trade-offs remain under discussion [2]. Screening men over 70 is identified by multiple bodies as a low-value activity for a variety of rationales including lack of clinical and economic value [6]. Current advice in the UK is that PSA testing should not be offered as a national screening programme [7] but men are advised to assess their own risk in discussion with their primary care physicians and hence to make an informed choice about being tested [8].

The cost-effectiveness of prostate cancer screening based on population-wide PSA testing is also uncertain, although it is known that costs of treating prostate cancer are high [9]. The Finnish Randomised Study of Screening for Prostate Cancer (FinRSPC), part of the European Randomised Study of Screening for Prostate Cancer (ERSPC) [10], followed up over 80,000 men for 20 years, but concluded that, when overall mortality was considered, neither arm could be defined as cost-effective [11]. As policy makers are interested in costs and benefits over a whole life time horizon in a screening context (particularly for prostate cancer which can take many years to manifest symptoms, if at all, during a man’s natural lifetime [12]), modelling approaches to deriving estimates of value for money are typically preferred over trial-based analyses for decision-making purposes. A recent systematic review of decision-analytical models designed to assess the cost-effectiveness of prostate cancer screening programmes demonstrated that there was substantial variation in model structure among the 10 included studies [13]. Potential cost-effectiveness was suggested in some of the studies under some screening strategies (*e.g.* annual screening starting at 55 years of age), but the lack of consistency in PSA threshold levels for further diagnostic investigation, invited age range, frequency of screen, modelling methodology, geographical location, progression pathway and optimal treatment precluded definitive conclusions from being drawn from these models.

Budget impact analysis (BIA) complements cost-effectiveness analysis by assessing the affordability of investing in a new intervention at the population level [14]. In the context of commercially provided technologies (*e.g.* a new drug or device) in the UK, the National Institute of Health and Care Excellence (NICE) specifies that a budget impact on the NHS of higher than £20 million (over and above existing costs) in one or more of the first three years of implementation is significant, and should lead to delayed implementation alongside further discussions with manufacturers [15]. The period of interest in a BIA model (on an annual basis) is typically the first 3 to 5 years of a new intervention, with the analysis considering the probable size of the population affected as well as the costs of both the intervention and any associated healthcare accessed by the patient within that period. Randomised controlled trials offer an ideal vehicle for determining short-term cost differences between patients given different treatments. For example, Klein et al. applied measured trial-based cost differences in depression treatments to estimate their potential budgetary impact [16].

The UK CAP trial compared a group of men aged 50–69 invited to take a single PSA screening test with a group of men offered usual care only [7] to determine effectiveness and cost-effectiveness of a single PSA test (equivalent to a prevalence screen). The primary clinical outcome of the trial was prostate cancer mortality after a median follow-up of 10 years. The study detected little evidence of a clinically important difference in prostate cancer mortality [4]. Nested within the intervention arm was the ProtecT three-arm treatment trial comparing active monitoring with radical prostatectomy and radical conformal radiotherapy. ProtecT found no evidence of a difference between the treatments in terms of prostate cancer mortality [17], but more metastases in the active monitoring group compared with the radical groups. A within-trial economic analysis suggested that costs and benefits were very similar although radiotherapy was more likely to be the cost-effective treatment option, with a 58% probability of being cost-effective at a typical UK willingness-to-pay threshold of £20,000 per QALY [18]. A lifetime decision model extrapolating the ProtecT data suggested, again with considerable uncertainty, that both radiotherapy and prostatectomy could be cost-effective in low risk populations, while prostatectomy was cost-effective in higher risk populations [19].

As far as we are aware, no study to date has looked specifically at the potential budget impact of prostate cancer screening using PSA testing in a UK context. The detailed resource-use data available at the individual patient level in CAP allow us to conduct a direct assessment of the potential impact on secondary care costs associated with introducing a population wide single PSA-based screening programme.

## Methods

### The CAP trial

Full details of the CAP trial methods (ISRCTN92187251) are available elsewhere [20]. Briefly, the trial is a pragmatic block cluster-randomised two-arm trial of a single invitation to prostate-specific antigen (PSA) testing to screen for prostate cancer, with long-term follow-up for all-cause and prostate cancer specific mortality. The trial was approved by Trent MREC [05/MRE04/78] and the Confidentiality Advisory Group [PIAG 1–05(f)/2006] [20]. Between 2001 and 2009, an invitation to take a PSA screening test was sent to each man aged 50–69 who was registered with GP practices randomised to the intervention arm. Men in the comparison arm practices were provided with usual care (*i.e.*, relevant information was provided to any man explicitly asking for advice about PSA testing, as later described under the guidance of the UK Prostate Cancer Risk Management Programme [7]).

### Study population

The study populations for the budget impact analysis were drawn from men aged 50–69 years registered at participating GP practices within the 8 trial centres in England and Wales (Sheffield, Newcastle, Bristol, Cardiff, Birmingham, Leicester, Cambridge and Leeds). Men were excluded from CAP if they had a prostate cancer diagnosis prior to randomisation or they opted out. Little evidence was found of baseline differences between the GP practices who consented to participate in the intervention compared with control practices [20], indicating the success of randomisation.

### Measurement of resource use

The analysis was conducted from the perspective of the UK NHS (secondary care) and related to the key cost drivers only (inpatient stays, day case visits and outpatient visits, for any reason) for this population. Resource use was measured through routinely collected data, validated for this purpose in previous work [21, 22]. Hospital Episode Statistics (HES [23]) from NHS Digital and Patient Episode Database for Wales (PEDW [24]) were used for men in England and Wales, respectively. HES and PEDW data were held by the Secure Anonymised Information Linkage (SAIL) Gateway [25] based in Swansea University alongside study identifiers and outcome measurements supplied by the CAP study team. Use of a trusted third party ensured that information governance requirements from the Confidentiality Advisory Group (CAG) for section  251 access to these data were fulfilled. Linkage of HES and PEDW data with trial data was carried out via SAIL, resulting in a pseudo-anonymised dataset that was analysed remotely via a secure remote desktop. Linkage was based on a combination of NHS number, date of birth, sex and postcode; 99.85% of men were successfully linked [4]. Hospital events recorded in both HES and PEDW were deduplicated using Stata functionality. The analysis used available resource-use data on all outpatient events, day cases and inpatient stays covering a period of 5 years from randomisation (the date on which the GP practice identified the list of men eligible to participate, referred to as the ‘list date’).

### Classification of events

Each record in the admitted patient care datasets represents a fixed consultant episode (the total time a patient spends under the care of an individual consultant). Long-stay inpatient events (defined as events lasting for over one year) were excluded from the analysis as they were indicative of residential care. Events were treated as day cases if they were classified as such in the patient class field or if they had stay lengths of zero nights.

We assigned Healthcare Resource Group (HRG) codes using the NHS Reference Costs Grouper [26] for both HES data and PEDW data. An HRG represents a group of patient events that have been judged to consume a similar level of resource. As adjustments to the HRG system are made on an annual basis, the 2013/14 Grouper (five years after the most recent list date) was used to ensure that as many codes as possible were still relevant. OPCS codes (which define the procedures and interventions that a patient has undergone while in hospital [27]) from earlier years that were no longer used were manually adjusted to the closest contemporary code. Any nights beyond the ‘trim point’ (defined for each HRG as the length of stay at the third quartile plus 1.5 times the inter-quartile range) were identified as excess nights for costing purposes.

The HES and PEDW outpatient datasets contain information on outpatient procedures and other outpatient visits. HRGs were also assigned through the 2013/14 Grouper, based on procedures where applicable.

### Application of costs

We applied unit costs from the UK Department of Health annual National Reference Costs (2013/2014) [28] to both English and Welsh data, adopting a fully pooled one country costing approach [29].

For the admitted patient care episodes, a cost for the relevant type of event (day case, elective, non-elective short stay or non-elective long stay) was matched to the HRG. The grouper software assigned UZ01Z error codes to events for which it was not possible to assign an HRG. UZ01Z costs were first published in (2014/15) [30]; a weighted average of these costs for each type of event was derived.

For procedure-driven outpatient events, unit costs were matched to the appropriate HRG. For all other outpatient events, information in relation to the main specialty and the type of medical staff was used to attach relevant unit costs. Missing values of the main specialty, or codes indicating ‘not a treatment function’ were assigned a General Medicine speciality code (300). Outpatient events that were assigned UZ01Z codes were treated as if they were the most common HRG (WF01A: Non-Admitted Face to Face Attendance, Follow-up).

Where appropriate costs were missing from the Reference Costs, weighted means of similar events were used. All costs were inflated to 2020 costs (the most recently available year) using the NHS cost inflation index [31]. The total cost for each individual man per year from randomisation was calculated as the sum of the costs of resource-use items.

### Budget impact analysis

We conducted a budget impact analysis at a population level to compare the average secondary care costs associated with all men in the two arms of CAP (i.e. an intention-to-treat analysis on all men in the trial, whether or not diagnosed with prostate cancer) to give an estimate of the likely budgetary impact to hospitals of introducing a single PSA-based screening programme in the UK over a time horizon of five years. The budget impact analysis adhered to relevant guidelines developed by the International Society for PharmacoEconomics and Outcomes Research (ISPOR) [14]. The analysis was conducted using Stata 16.1 [32].

A simple cost calculator approach was taken, based on the observed intention-to-treat differences in costs between the two trial arms. The two groups were compared as randomised on an intention-to-treat basis using a multi-level modelling approach, incorporating both cluster and practice levels. As the budget impact analysis aims to estimate the actual impact at each time point rather than the perceived value placed on the investment, costs were not discounted, in line with good practice principles [14].

Cost differences between the arms were extrapolated to the population eligible for screening based on population estimates from the Office of National Statistics for England and Wales [33]. Uptake of the PSA invitation in the CAP intervention arm was low at 40% [4]. Therefore, an overall budget impact was also estimated for a possible uptake rate of 80% for a newly rolled out screening programme, based on the uptake in men, who are of a comparable age, of a screening programme for abdominal aortic aneurysm [34, 35]. A linear relationship between the cost and uptake was assumed.

### Sensitivity analyses

Sensitivity analyses were used to explore the effect of methodological uncertainty or assumptions made during the course of the study and analysis. As the HES outpatient dataset did not exist prior to 2003, and was seen as an experimental dataset from 2003 to 2008 [36], a sensitivity analysis was conducted on HES and PEDW inpatient data only. It is not possible to accurately identify episodes relating to prostate cancer because of the limited diagnosis information in the outpatient dataset; however, a sensitivity analysis was conducted restricting the episodes considered to those associated with urology, using the HRG codes for the inpatient dataset and the specialty for the outpatient dataset (urology = 101). As the CAP trial was based on a single invitation for PSA screening, no information was available on second and subsequent screening invitations. A sensitivity analysis considered a one-off screen at age 55 only [37]; age at randomisation was calculated from the month and year of date of birth, assuming that the day was the 15th.

## Results

### Population

**Fig. 1:**
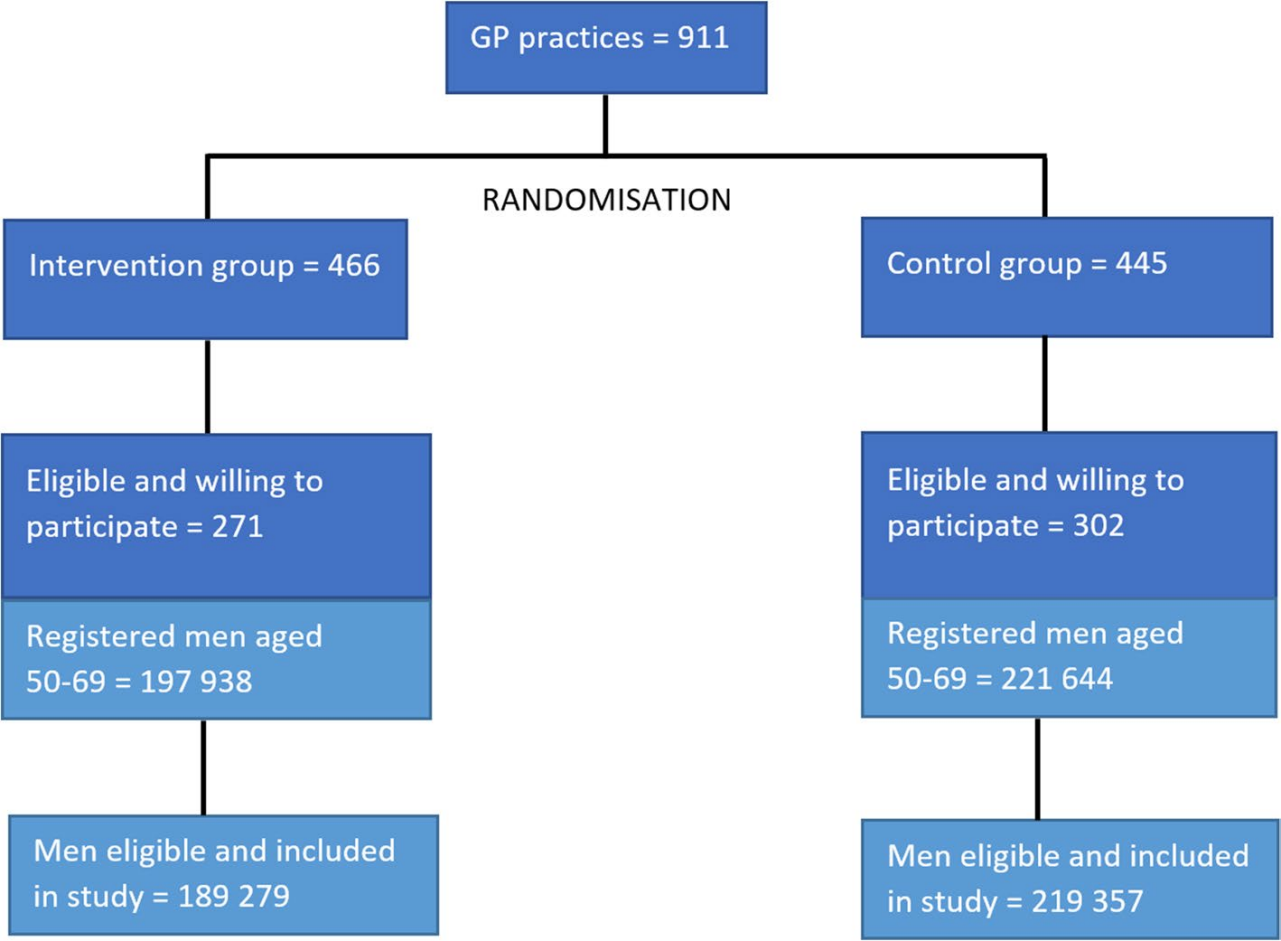
Flow of men through the study

Practices were randomised between 2001 and 2009, resulting in 189,279 men randomised to the intervention arm to receive an invitation to take a PSA test and 219,357 men randomised to receive usual NHS care. The flow of men through the study is depicted in Fig. 1.

### Resource use

Over the five-year period of interest, the NHS grouper software assigned error codes to 28832 (3.2%) inpatient stays and 1732 (0.057%) outpatient appointments. The 10 most common inpatient HRGs for any reason are given in Table 1 with associated unit costs, showing that dialysis, chemotherapy and diagnostic flexible cystoscopy were the commonest inpatient encounters for this male population.

**Table 1:** Commonest inpatient HRGs encountered in the analysis, combining costs directly due to prostate cancer and non-prostate cancer costs

**HRG**	**HRG description**	**Number of events**	**Unit costs (2020 £)**			
			**Day case**	**Elective inpatient**	**Non-elective inpatient long stay**	**Non-elective inpatient short stay**
LA97A^a^	Same Day Dialysis Admission or Attendance, 19 years and over	94877	-	-	-	-
SB97Z^a^	Same Day Chemotherapy Admission or Attendance	29077	-	-	-	-
LB72A	Diagnostic Flexible Cystoscopy, 19 years and over	24073	£455	£1225	£7026	£834
FZ61Z	Diagnostic Endoscopic Upper Gastrointestinal Tract Procedures with Biopsy, 19 years and over	21398	£489	£940	£3127	£535
EB12C	Unspecified Chest Pain with CC Score 0–4	18424	£552	£739	£1167	£433
SC97Z^a^	Same Day External Beam Radiotherapy Admission or Attendance	13588	-	-	-	-
BZ02C	Phacoemulsification Cataract Extraction and Lens Implant, with CC Score 0–1	12476	£942	£1361	£2471	£1365
LB27Z	Minor Endoscopic, Prostate or Bladder Neck Procedures (Male)	£12084	£740	£1687	£6112	£697
FZ60Z	Diagnostic Endoscopic Upper Gastrointestinal Tract Procedures, 19 years and over	£11491	£448	£895	£2492	£557
FZ92K	Malignant Gastrointestinal Tract Disorders without Interventions, with CC Score 0–2	£11311	£395	£1877	£2216	£594

*CC* Complications/comorbidities

^a^These HRGs attract zero costs themselves, with ‘unbundled’ HRG costs added on

Table 2 gives the resources used most commonly by intervention vs control arm. The commonest inpatient events did not differ between arms, except for Minor Endoscopic, Prostate or Bladder Neck Procedures (LB27Z) and radiotherapy (SC97Z), both higher in the intervention arm. For outpatient events, both consultant-led (0.0169, *p* < 0.001) and non-consultant-led (0.0331, *p* < 0.001) urology appointments were significantly higher in the intervention arm, while other non-consultant-led appointments were slightly lower (-0.0478, *p* < 0.001). The procedure-driven outpatient appointments differed by 0.0009 events between arms (*p* = 0.04) for minor skin procedures (JC43A).

**Table 2:** Comparison of population mean (sd) unadjusted NHS secondary healthcare resource utilisation by trial arm in the first year following randomisation

**Resourc**e	**Mean number of events in intervention arm** **(*n* = 189,279)**	**SD intervention**	**Mean number of events in control arm** **(*n* = 219,357)**	**SD control**	**Difference between arms**	***p*-value**
**Inpatient**						
BZ02C (Phacoemulsification Cataract Extraction and Lens Implant, with CC Score 0–1)	0.005	0.077	0.005	0.081	0.000	0.073
EB12C (Unspecified Chest Pain with CC Score 0–4)	0.010	0.138	0.009	0.126	0.001	0.154
FZ60Z (Diagnostic Endoscopic Upper Gastrointestinal Tract Procedures, 19 years and over)	0.006	0.083	0.006	0.079	0.000	0.973
FZ61Z (Diagnostic Endoscopic Upper Gastrointestinal Tract Procedures with Biopsy, 19 years and over)	0.010	0.106	0.010	0.107	0.000	0.558
FZ92K (Malignant Gastrointestinal Tract Disorders without Interventions, with CC Score 0–2)	0.009	0.359	0.009	0.373	0.000	0.786
LA97A (Same Day Dialysis Admission or Attendance, 19 years and over)	0.044	2.405	0.041	2.291	0.003	0.666
LB27Z (Minor Endoscopic, Prostate or Bladder Neck Procedures (Male))	0.025	0.186	0.003	0.060	0.022	0.000
LB72A (Diagnostic Flexible Cystoscopy, 19 years and over)	0.011	0.118	0.011	0.116	0.000	0.693
SB97Z (Same Day Chemotherapy Admission or Attendance)	0.005	0.227	0.005	0.213	0.000	0.964
SC97Z (Same Day External Beam Radiotherapy Admission or Attendance)	0.004	0.340	0.001	0.141	0.003	0.000
**Outpatient**						
Urology						
Non-consultant led	0.054	0.299	0.020	0.230	0.0331	< 0.001
Consultant led	0.075	0.429	0.058	0.379	0.0169	< 0.001
Medical Oncology						
Non-consultant led	0.001	0.064	0.001	0.123	-0.0007	0.02
Consultant led	0.011	0.286	0.010	0.265	0.0008	0.33
Palliative						
Non-consultant led	0.000	0.026	0.000	0.014	0.0001	0.43
Consultant led	0.001	0.069	0.001	0.088	0.0003	0.21
Other						
Non-consultant led	0.224	1.145	0.272	1.318	-0.0478	< 0.001
Consultant led	0.856	2.294	0.866	2.300	-0.0098	0.17
Procedure-driven outpatient appointments						
LB72A (Diagnostic Flexible Cystoscopy, 19 years and over)	0.001	0.039	0.001	0.039	0.0000	0.90
JC43A (Minor Skin Procedures, 13 years and over)	0.003	0.117	0.004	0.155	-0.0009	0.04
Other procedure-driven appointments	0.014	0.202	0.013	0.167	0.0003	0.65

### Costs and budget impact

Mean population-level NHS secondary care costs in all trial men by arm, and cost differences comparing all men invited for a single PSA screening test vs all men in the control arm are given on an annual basis in Table 3. Costs differed significantly for the first year following randomisation (£44.80 higher in the intervention arm, 95%CI: £18.30 to £71.30), but not for the subsequent years, suggesting that treatment was often carried out promptly. In the first year following randomisation, the budget impact on NHS secondary care in England and Wales (based on the uptake of the PSA screening test observed in the CAP trial) was estimated to be up to £314 million. If uptake of the PSA screening test had been nearer 80%, the budget impact on NHS secondary care could have potentially been £628 million.

**Table 3:** Comparison of mean costs (95% CI) between trial arms, by year since randomisation (2020 £)

	**Mean cost intervention arm** **(*n* = 189279)**	**Mean cost control arm** **(*n* = 219357)**	**Within trial cost difference**	**Population**^**a**^** level budget impact (£million)**	***p* value**
**Year 1**	812 (798 to 826)	763 (751 to 776)	44.8 (18.3 to 71.3)	314 (128 to 500)	0.001
**Year 2**	851 (837 to 866)	835 (821 to 848)	13.0 (-12.2 to 38.2)	91 (-85 to 268)	0.3
**Year 3**	888 (873 to 903)	882 (868 to 895)	0.9 (-25.4 to 27.3)	7 (-178 to 191)	0.9
**Year 4**	932 (916 to 947)	944 (930 to 959)	-15.4 (-43.4 to 12.5)	-108 (-304 to 88)	0.3
**Year 5**	974 (958 to 990)	992 (977 to 1007)	-22.8 (-51.8 to 6.2)	-160 (-363 to 44)	0.1
**First 5 years**	4457 (4412 to 4501)	4416 (4375 to 4456)	12.6 (-92.5 to 117.7)	88 (-649 to 825)	0.8

^a^7,012,201 men aged 59 to 65 in England and Wales [33]

### Sensitivity analyses

Cost differences in the first year following randomisation, and the first 5 years after randomisation, for each of the sensitivity analysis scenarios are given in Table 4. The first-year difference observed in the primary analysis was retained in the sensitivity analyses based on inpatient data only and on urology events, although the urology events analysis also suggested that the difference persisted through the first 5 years overall in contrast to the base case. No significant difference was observed when the analysis is based only on men aged 55 at randomisation.

**Table 4:** Results of sensitivity analyses by trial arm in first year and first 5 years following randomisation

	**Mean cost intervention arm (95%CI)**	**Mean cost control arm (95%CI)**	**Within trial cost difference (95%CI)**	**Population**^**a**^** level budget impact (95%CI) (£million)**	***p* value**
Inpatient data only	*n* = 189279	*n* = 219357			
Year 1	646 (633 to 659)	596 (585 to 608)	44.6 (22.0 to 67.3)	313 (154 to 472)	< 0.001
First 5 years	3484 (3443 to 3524)	3435 (3399 to 3472)	17.4 (-70.7 to 105.4)	122 (-495 to 739)	0.7
Urology events only	*n* = 189279	*n* = 219357			
Year 1	82 (79 to 85)	50 (48 to 52)	31.8 (27.5 to 36.1)	223 (193 to 253)	< 0.001
First 5 years	339 (332 to 346)	299 (293 to 306)	38.5 (26.4 to 50.6)	270 (185 to 355)	< 0.001
Restricted to age 55	*n* = 11123	*n* = 12454			
Year 1	590 (542 to 638)	593 (549 to 636)	-3.6 (-68.2 to 60.9)	-26 (-478 to 427)	0.9
First 5 years	3360 (3210 to 3510)	3426 (3278 to 3575)	-78.7 (-295.8 to 138.4)	-552 (-2074 to 970)	0.5

^a^7,012,201 men aged 59 to 65 and 405,400 men aged 55 in England and Wales [33]

## Discussion

### Summary of results

This study has indicated that there could be substantial costs associated with the early years of a PSA testing programme for detecting prostate cancer. If all men aged 50–69 in England and Wales were to be offered the test simultaneously, the associated NHS secondary care costs arising from treatment of detected cancers could run to £628 million, which is unlikely to be affordable in the UK context, given NICE considerations [15]. The abdominal aortic aneurysm screening programme was introduced in phases [38], and it is more likely that a subset of men would be involved in any prostate cancer programme initially. The sensitivity analyses mostly support the main conclusions, although restricting the sample to age 55 only suggests that there is some uncertainty when smaller groups are considered.

The results reflect that men diagnosed in the screening arm were treated promptly, leading to the observed higher costs in the first year. In subsequent years without the intervention, the number of men accessing treatment may have been more evenly balanced, so that it was not possible to detect differences above the general healthcare that men aged 50–69 receive. The observed costs increase as the years progress, which is likely to be due to the men requiring more treatments (for any reason) as they age.

### Study strengths and weaknesses

The study involved a large sample of men analysed at an individual patient level, and the benefits of randomisation were preserved as men were analysed on an intention-to-screen basis. Resource use was measured using routine data validated for prostate cancer research purposes [21, 22] and covered all causes, which prevented attribution bias and ensured that treatments arising from complications were taken into account (*e.g.* heart issues associated with prostate cancer treatments). The use of all-cause resource-use data strengthened our conclusions, as a difference between the groups was detected despite the ‘noise’ of other contacts.

However, there are also some limitations. A very small number of men who were included in the primary CAP analysis [4] were excluded from this analysis due to the anonymisation process preventing accurate identification of men who received a diagnosis or died within the first month after randomisation. Changes have occurred in the management of both prostate cancer and the NHS itself since data collection began. For example, more recent innovations have included the use of new biomarkers, and MRI-guided biopsy methods; model-based economic evaluations have suggested that these methods may be more cost-effective [39]. Costs associated with the intervention arm may not accurately reflect the resources used or the time to treatment experienced in normal practice, as participants were randomised to one of three treatments as part of the embedded ProtecT treatment trial [18], and there is now increased use of active surveillance for low risk disease. Men involved in the ProtecT treatment trial who were diagnosed with localised prostate cancer would have had prompt and enhanced follow-up in all treatment arms; the effect of this on secondary care costs is uncertain. Radiotherapy was not routinely recorded in HES data prior to 2011, which may have led to underestimates of the costs associated with the nested radiotherapy arm in the CAP intervention arm. This analysis was conducted from the secondary care perspective, but it was not possible to include data from Accident and Emergency (A&E) visits; however, we do not anticipate any important differences in A&E costs between arms and the conclusions are, therefore, unlikely to be altered. Using routinely collected data meant that a number of assumptions had to be made (detailed in the Methods section) with regards to selecting the resource use and applying appropriate unit costs. It is possible that there was some mis-coding of procedures in the HES and PEDW data; a study looking at urological events in 2012 concluded that approximately 20% of procedures were coded with errors [40]. However, there is no reason to believe that errors are more likely in one arm than the other.

### Comparison with other research

Budget impact analysis methodology has been applied to several cancer-screening programmes. A systematic review identified 19 such studies (the majority of which were based on decision-analytic models [41]), but found poor adherence to guidelines (e.g. [14]). Only three studies considered prostate cancer screening programmes, none of which were based purely on PSA testing; one looked at the cost of using prostate cancer antigen 3 (PCA3) urine testing [42], and the other two considered risk scoring approaches [43, 44]. A US-based study found that the budgetary impact of an ongoing PSA testing regime (annually for men aged 66 to 99) was substantial for the government-funded Medicare population, with a national estimate of over $450 million per annum [45]. A decision-analytic modelling approach illustrated that the costs associated with screening (including screening, diagnosis, treatment and complications) were higher for older men in the US context, suggesting that targeting screening could reduce the budgetary impact [46].

### Implications for policy makers

Policy makers must consider whether screening programmes are affordable within the budget available. We have shown in the case of PSA testing for prostate cancer that this is potentially questionable in the UK context. However, a budget impact analysis does not supply evidence about the value for money that the programme offers, so policy makers should consider our results alongside effectiveness and cost-effectiveness evidence [47]. Even when restricted to secondary care only, the costs to the NHS of implementing a screening programme are potentially substantial. The patient population (in both arms) may also have consumed considerable resources, particularly in the end of life period [48–50], in terms of hospice care and primary care [51], and substantial costs may accrue to the public sector more generally for care needs. In addition, the costs of the screening programme and subsequent diagnosis (including staff training, quality assurance, audit and national administration as well as the testing costs) would need to be considered [52].

### Future research

While neither the CAP trial [4] nor a wider systematic review and meta-analysis [5] of PSA testing for prostate cancer found evidence of effectiveness for all-cause mortality, it is possible that cost-effectiveness studies may reach seemingly paradoxical results in favour of implementation [53]. Recent work incorporated the measurements made in the CAP trial into a cost-effectiveness model to supply evidence of value for money, finding that a one-off screen at 50 years of age was potentially cost-effective [47]; following up men to a median of 15 years (currently underway) may reduce uncertainty in the cost-effectiveness estimates. Future research should focus on assessing whether the ongoing costs of treatment arising from a screening programme meet affordability criteria across all sectors.

## Conclusions

The introduction of a PSA testing programme for prostate cancer has the potential to have a substantial budgetary impact on hospital care, based on data from a large randomised controlled trial carried out in the UK. Decision makers wanting to implement such a programme should consider whether it is affordable within local budgetary constraints, and take affordability (based on realistic contemporary cost estimates) into account alongside measures of effectiveness and cost-effectiveness.

## Data Availability

The data used in this study were provided by NHS Digital and we are not permitted to share them. Access requests for HES data can be made through NHS Digital (https://digital.nhs.uk/services/data-access-request-service-dars/dars-guidance). For data collected within the CAP trial researchers can apply to access these data where appropriate governance is in place by contacting Dr Turner in the first instance (emma.turner@bristol.ac.uk).
